# Composite alginate gels for tunable cellular microenvironment mechanics

**DOI:** 10.1038/srep30854

**Published:** 2016-08-03

**Authors:** Adele Khavari, Magnus Nydén, David A. Weitz, Allen J. Ehrlicher

**Affiliations:** 1 Applied Chemistry, Chemical and Biological Engineering, Chalmers University of Technology, SE-412 96 Göteborg, Sweden; 2 SUMO Biomaterials VINN Excellence Center, Chalmers University of Technology, SE-412 96 Göteborg, Sweden; 3 UCL Australia, 220 Victoria Square, Adelaide, SA 5000 Australia; 4 Department of Bioengineering, McGill University, Montreal Canada H3A 0C3; 5 School of Engineering and Applied Sciences, Department of Physics, Harvard University, Cambridge, Massachusetts 02138, USA; 6 Beth Israel Deaconess Medical Center, Boston, Massachusetts 02115, United States; 7 Harvard Medical School, Boston, Massachusetts 02115, United States

## Abstract

The mechanics of the cellular microenvironment can be as critical as biochemistry in directing cell behavior. Many commonly utilized materials derived from extra-cellular-matrix create excellent scaffolds for cell growth, however, evaluating the relative mechanical and biochemical effects independently in 3D environments has been difficult in frequently used biopolymer matrices. Here we present 3D sodium alginate hydrogel microenvironments over a physiological range of stiffness (E = 1.85 to 5.29 kPa), with and without RGD binding sites or collagen fibers. We use confocal microscopy to measure the growth of multi-cellular aggregates (MCAs), of increasing metastatic potential in different elastic moduli of hydrogels, with and without binding factors. We find that the hydrogel stiffness regulates the growth and morphology of these cell clusters; MCAs grow larger and faster in the more rigid environments similar to cancerous breast tissue (E = 4–12 kPa) as compared to healthy tissue (E = 0.4–2 kpa). Adding binding factors from collagen and RGD peptides increases growth rates, and change maximum MCA sizes. These findings demonstrate the utility of these independently tunable mechanical/biochemistry gels, and that mechanical confinement in stiffer microenvironments may increase cell proliferation.

It has become increasingly apparent that forces and mechanics directly impact cell function in many diverse contexts, from cardiovascular remodeling to stem cell differentiation, and mechanobiology is likely an essential aspect of pathology. In cancer and metastasis, key physical changes occur both in the microenvironment and in the cells themselves^1^. *In vivo*, the microenvironment surrounding a cancerous cell changes and typically becomes stiffer as the tumor progress^2^,^3^, often due to collagen remodeling and crosslinking^4^,^5^. In the case of breast cancer, the elastic moduli of breast tissues range from 0.4 to 2 kPa in a normal state, and increase by about an order of magnitude to 4–12 kPa in the cancerous state^3^,^6^. This matrix stiffness directly influences cytoskeleton mechanics^7^; stiff substrates specifically increase cytoskeleton tension, which in turn promotes both cell proliferation^8^,^9^,^10^ and directional movement^11^,^12^. While in the progression of cancer from benign, to carcinoma, to metastatic the microenvironment typically stiffens, conversely the cells themselves become softer^13^, a trend that has been observed with a broad variety of cancers^14^. In addition to cells becoming softer with increasing metastatic potential, the acto-myosin network is more contractile, and exerts larger forces^15^,^16^,^17^. This combination of microenvironment stiffening, cellular softening, and increased cellular traction forces create a physical state which strongly favors cell dissemination, a key part of metastasis.

While these mechanobiology relationships have been frequently investigated in isolated cells on substrates, studying cell dynamics in three-dimensions has been a persistent challenge; there are obvious difficulties imaging cells in gels, as well as maintaining cell viability over the scale of weeks. The composition of the gels themselves is also a complication, as it is difficult to independently investigate mechanics and biochemistry in scaffolds derived from extra-cellular matrix as changing the components simultaneously effects chemistry and mechanics. Three-dimensional studies are often performed in gels assembled from polymers such as collagen^18^ or fibrin^19^, which while excellent physiological models, are not biochemically inert, making it difficult to discriminate between mechanical and chemical effects in cell behavior. An ideal matrix for studying mechanobiology would have independently tunable mechanical and chemical properties, and be capable of spanning physiologically relevant stiffness ranges. Agarose has been used to create 3D cell cultures^20^, however, agarose gels are difficult to mechanically control, and are sensitive to numerous factors such as time and temperature of gelling, and the molecular distribution of the polymer; these challenges of agarose make it difficult to create substrates with precise physiological stiffnesses^21^,^22^. Sodium alginate is a unique alternative network material, which can be formed at 37 °C at neutral pH, can be modified to include RGD peptide binding sites^23^, or copolymerized with collagen^24^,^25^ to create an interpenetrating network of alginate and collagen to investigate the influence of mechanics on the progression of wound healing. Previously the collagen/alginate combination has been used to create a structurally switchable 3D extracellular matrix^26^. As a polymer base for cell culture, alginate has many favorable attributes, including biocompatibility, lack of immunoresponse, precise mechanical control, no mammalian receptors, and low protein adsorption^27^,^28^.

In this article we fabricate 3D cellular scaffolds using combinations of alginate, RGD, and collagen to create precisely mechanically controlled gels with shear moduli from 1.85 to 5.29 kPa, with or without biochemical binding factors. We then use these gels to examine the growth of multicellular aggregates (MCAs) that serve as model tumors of progressively metastatic murine cell lines^17^,^29^. We find that the growth and morphology of these MCAs is set by the external microenvironment stiffness, and that MCAs grow larger and faster in stiffer environments. Binding factors from collagen or RGD peptides increase growth rates, but do not significantly change maximum MCA sizes. These findings demonstrate that mechanical confinement in stiff microenvironments may actually promote growth of cell clusters. Understanding these effects may in the future provide new strategies for diagnosing or even treating cancers by targeting mechanical changes.

## Materials and Methods

### Alginate preparation and characterization

A stock alginate solution of 2.5% by weight was prepared by adding CO_2_-independent growth medium (Invitrogen, USA) to sterile alginate with an average molecular weight between 150–250 kDa (Pronova SLG100, FMC BioPolymer, Norway). CO_2_-independent media was selected as it allows longer imaging sessions out of incubation, and because gels formulated with this media were measured to have more consistent rheological properties. The solution was placed in 37 °C water bath for 1 hour and then vortexed for 30 min in room temperature to dissolve completely. To make a homogenous alginate gel^30^, a nanoparticle calcium carbonate (mkNANO, Canada) suspension in MQ-water was used as a Ca^2+^ source in combination with the slowly hydrolyzing D-Glucono-δ-lactone(GDL) (Sigma-Aldrich Co), which releases the Ca^2+^ in the solution. The calcium carbonate suspension was mixed vigorously with three different concentrations of alginate to make gels with final concentrations of 0.5, 1, and 2 w/v %. To retain the same chemical environment for all the samples, the calcium carbonate and GDL concentrations were chosen as such to reach a final concentration of 30 mM and 60 mM, respectively. The concentration of GDL was always kept at twice that of the calcium carbonate, the stoichiometric ratio for the GDL and CaCo_3_ reaction, resulting in a pH of 7–7.5 in the solution^28^.

Prior to gel formation, the mixture was sonicated for 8–10 minutes to de-gas, and then immediately used for mechanical measurements, or mixed with cells for encapsulation. To include an adhesive peptide for some substrates, RGD-alginate (NOVATACH G RGD GRGDSP-coupled high G alginate, FMC BioPolymer, Norway) was dissolved in CO_2_-independent growth medium and mixed with the same three different alginate concentrations as above to obtain a final concentration of 100 fmol/ml of the peptide in each sample. For alginate/collagen blends, alginate and collagen (PureCol, Advanced BioMatrix, USA) solutions were formulated to reach the final concentration of 0.75 w/v % and 0.1 mg/ml (3.3 × 10^8^  fmol/mol), respectively. These concentrations of binding factors were chosen to retain the same mechanical properties as the highest concentration of pure alginate. Both alginate/collagen and alginate/RGD-alginate blends were gelled with 30 mM calcium carbonate and 60 mM GDL with the same procedure as the pure alginate gel. The CO_2_- independent medium used to make all the solutions was supplemented with 10% fetal bovine serum (FBS, Invitrogen, USA) and 1% antibiotics (Pen/strep Invitrogen). The alginate pores size at the concentrations used in this study are between 5 nm to 200 nm, as reported in previous studies^27^,^31^. This pore size and the hydrophilic nature of alginate gels allow proteins and nutrient molecules to diffuse freely in the gel^32^,^33^,^34^.

Rheological properties of all the samples were measured in oscillation mode using an AR-G2 rheometer (TA Instruments). Time dependence of the dynamic storage modulus (shear modulus) was measured during gelation. The precursors were placed between a 25 mm cone and plate geometry, and sealed with silicone oil to avoid water evaporation during measurements. Applied strain and frequency were fixed at 0.5% and 1 Hz, respectively. Dynamic frequency sweep experiments were carried out from 0.1 to 100 Hz after the gelation with a fixed strain at 0.5%, which is in the linear viscoelastic region, and these mechanical measurements are summarized in Supplementary Fig. S4.

The pH of each system was determined at room temperature, and after incubating at 37 °C, using pH strips. The pH of all the formulations was in the range of 7–7.5 before and 6.5–7 after incubation in 37 °C, 95% humidity, 95% air, and 5% CO_2_ atmosphere for 2 days.

### Cell culture in alginate gels

M28, M6, and M6c are three progressively metastatic mouse cancer cell lines, having been isolated from normal tissue, a local tumor, and a distal tumor, respectively, these cell lines has been developed by Holzer *et al*.^29^. M28 cells are benign or weakly tumorigenic, and were initially isolated from the healthy mammary gland of a 2-month-old female mouse. M6 cells, carcinoma, were initially isolated from a solid and homogenous mammary tumor on a 5 and 1/2-month-old female mouse. M6c cells are metastatic, and were derived from M6 cells which had migrated and metastasized in mouse lungs^29^. Cells were cultured with established techniques, then trypsinized, centrifuged, and dispersed in growth medium just prior to embedding in alginate. After sonicating the alginate precursors, dispersed cells were mixed in by gently pipetting up and down to avoid producing air bubbles. 350 μl of the mixture was transferred to each well of a 48-well plate, with approximately 2 × 10^5^ cells per well. The samples were incubated in cell culture incubators at 37 °C, 95% humidity, 95% air, and 5% CO_2_ atmosphere for 5 hours; after 5 hours when the samples had fully gelled, 350 μl of CO_2_-independent growth medium was added on top of the gel in each well and incubated in the same atmosphere. The medium was exchanged every 2 days.

### Cell transfection, fluorescence staining and imaging

M6 cells were transfected with Nuclear Location Sequence tagged with Green Fluorescent Protein (BD Biosciences, modified pEGF-C1 with NLS-GFP) using Lipofectamine 2000 (Invitrogen) following Invitrogen’s protocol. After transfection, successfully transfected cells were isolated by FACS (MoFlo XDP Cell Sorter), and growth of untransfected cells was suppressed by adding a selective antibiotic (G418, Invitrogen) to the growth medium. After three days of selection, the positively transfected cells were then resuspended in the 3D gel scaffolds. M28 and M6c cells were fluorescently labeled with green CMFDA (Invitrogen) after being encapsulated in the gel.

For all cells, images were captured every 2 to 5 days using a 10x objective on an inverted confocal microscope (Leica TCS SP5) with a programmable scanning stage. For each sample 4–6 images in different XY positions over a 1.29 mm by 1.29 mm area and Z depth of 1–2 mm, with a 10 μm Z step size were acquired. All images were analyzed using LAS AF software (Leica) and ImageJ.

## Results

### Mechanical characterization of alginate blend substrates

To create different mechanical and chemical environments for the cells, we prepared different matrices using alginate, collagen, and RGD-alginate (detailed in the Methods section). Gelation times (where the shear modulus becomes constant) for each sample were determined by the time sweep rheological measurements. It is crucial to know the gelation time for sample preparation to allow proper dispersal of the cells in the gel precursor before solidification. After complete gelation, shear moduli of the gels were measured with frequency sweep measurements in the linear elastic region where the strain is 0.5%. All measurements were performed in CO_2_-independent growth medium enriched with 10% FBS and 1% antibiotics, and are summarized in Table 1, and in detail in Supplementary Fig. S4.

The stiffness of the gels is increased by increasing the alginate concentration while keeping the calcium absolute concentration constant, and in all formulations there are sufficient calcium ions to percolate crosslinks within the alginate gel. Additionally, higher alginate concentration results in shorter gelation time, which ranges from 4–6 hours. Adding collagen to the above alginate concentration substantially increases the shear modulus; thus to have the same mechanical environment yet include collagen, the alginate concentration must be reduced to create a shear modulus similar to the pure alginate systems. Blending alginate with RGD-alginate had little effect on the shear moduli of the samples.

Rheology was performed at 23 °C to establish gelation times for sample preparation at room temperature. There is approximately a 10% increase in the stiffness of the alginate gels from 23 °C to 37 °C^35^, thus in culture the alginate gels are uniformly mildly stiffer than measured, and the moduli reported throughout the text reflect the stiffness at 37 °C as experienced by the cells. These alginate concentrations have been chosen to create moduli which approximate the physiological stiffness of the breast tissue both in normal/resting state of 0.4 to 2 kPa and pathological/activated state of 4–12 kPa^3^,^6^. Since the alginate is not enzymatically degradable in mammals^36^, the mechanical properties are constant over the course of the experiment. Additionally, cells did not appreciably degrade collagen in composite samples, as collagen fibers were imaged with confocal microscopy and appeared intact over 15 days culture (see Supplementary Fig. S4).

### Different cell lines respond differently to the surrounding scaffold mechanics

M28, M6, and M6c represent different stages of cancer progression from noninvasive (M28) to highly invasive (M6c), and are encapsulated within pure alginate of different moduli over the course of 7 days. As described above, M28 and M6c cells were stained with a fluorescence cell tracker and M6 cells were transfected with GFP and imaged with confocal microscopy every other day.

Over a week of observation, these different cell lines display very distinct growth characteristics; M28, the least invasive cells, do not appear to grow appreciably in any investigated gels or conditions. M6, tumorigenic but not invasive cells, grow faster than M28 or M6c in gels of all stiffnesses, with the fastest growth occurring in the intermediate stiffness gel of 2.85 kPa. M6c, metastatic cells, also display their fastest growth in the 2.85 kPa gel (see Supplementary Fig. S3).

**Figure 1:**
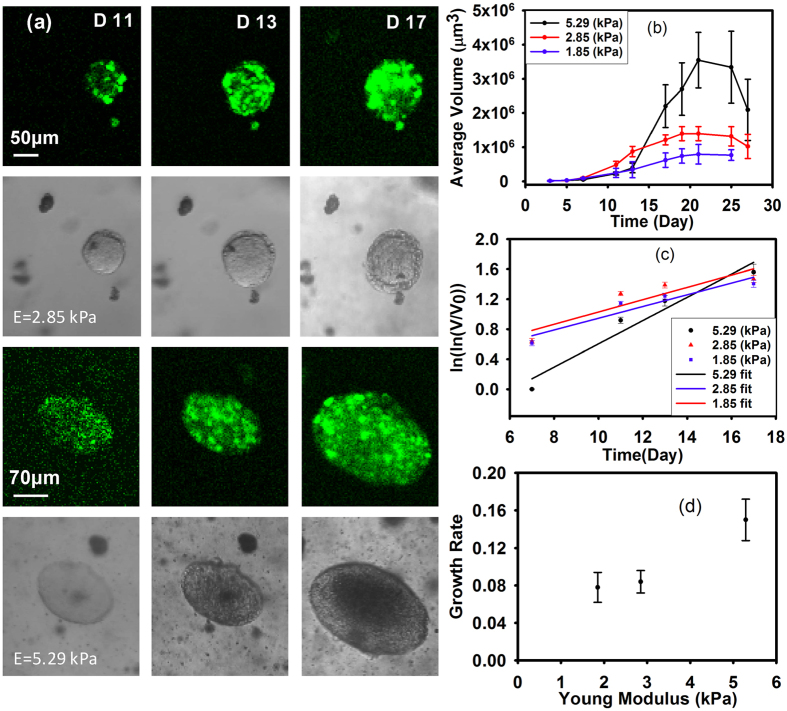
MCAs grow bigger in the stiffest gel (E = 5.29 kPa). (**a**) Fluorescence and phase contrast images of M6 MCA growth in two different stiffness gels over Days 11, 13 and 17, respectively. **Top panels;** in the intermediate stiffness gel (E = 2.85 kPa). **Bottom panels** MCAs grow bigger in the stiffest gel (E = 5.29 kPa). (**b**) Growth of M6 (carcinoma) cells within the gels of three different stiffnesses for long time (27 days). Means are shown from four separate experiments, including 40 to 100 MCAs at each time point for each culture condition. Error bars are standard error of the mean. For statistical analysis we did t-test or Mann-Whitney test depending on the data distribution for each time point and P-values are between 0.3–0.6 which shows there is not a statistically significant difference in the results. (**c**) Volume changes of the MCAs during time, before the growth plateau; the slope can be interpreted as MCA growth rate which is shown in (**d**) for different stiffnesses. The growth rate is dimensionless, and is the fastest for the stiffest matrix.

As the M6 cells grow fastest in all investigated stiffnesses, and were successfully stably transfected, we monitored M6 MCA’s growth for an extended duration of 27 days using confocal microscopy. We measured individual MCA ellipses, revealing their size distribution as a function of time, as shown in Supplementary Fig. S1. We also calculated their average volume in three different stiffnesses over time. The volume for MCAs was calculated as 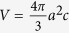, where *a* is the minor axis diameter and *c* is the major axis diameter, and these changes in volume over time are summarized in Fig. 1b. We find that the stiffer microenvironment matrices increase cell proliferation, and the average final sizes of MCAs are 200 (SD = 80), 158 (SD = 40), 112 (SD = 30) μm for 5.29, 2.85, 1.85 kPa gels, respectively.

Long time-scale behavior of the MCAs shows phases of growth lag, expansion, and shrinkage: MCAs in these scaffolds do not grow indefinitely larger, but appear to reach a maximum size, and then shrink somewhat, as shown in Fig. 1a. Until day 13, the MCAs in the 2.85 kPa gel are larger than in the ~1.85 kPa or ~5.29 kPa gels. After day 13, cells in the stiffest (5.29 kPa) gel dramatically increase their growth rate (Fig. 1e), while cells in the less stiff gels (1.85 and 2.85 kPa) seem to have reached a maximum size, and then appear to shrink, possibly due to cell death in the MCA centers.

To quantify the cellular growth rate in different mechanical environments, we applied the Gompertz law approach as described previously^37^. MCAs will initially grow slowly, then rapidly, and finally slowly. We can partition these phases into different linear stages of growth over time; in doing so, we can see that MCAs follow Gompertz’s law:


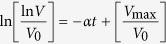


where V is the MCA volume, V_0_ initial volume, and V_max_ the final volume. The parameter α can be considered as the dimensionless growth rate. When we plot the data shown in Fig. 1b as ln(lnV/V_0_) vs. time, the characteristic growth rate becomes more apparent, and is shown in Fig. 1c.

The data from day 7 through day 19 (before the growth plateau phase) can be fit by a single linear fit by the best approximation (r^2^ = 0.93) using Table Curve (Systat Software). Values of parameter α, are 0.078, 0.084, 0.15 for 1.85, 2.85, and 5.29 kPa gels, respectively (see Fig. 1d).

Here, we can see a clear increase in alpha as a function of increasing gel stiffness.

In Fig. 1a, the top panel shows the gradual growth of a MCA in 2.85 kPa gel, demonstrating that the MCA growth has reached a plateau phase. The bottom series of images depict the dramatic increase (from 160 to 240 μm) in MCA size from day 13 to 17 in the stiffest gel (E = 5.29 kPa). These images are representative of typical behavior, and also illustrate the stark transition from spherical to elliptical MCAs in stiffer gels.

The underlying molecular mechanisms for the correlation between MCA or tumor growth and microenvironment stiffness are uncertain and likely complex, but may be related to mechanotransduction through cytoskeletal strain^38^,^39^,^40^, or microRNA expression which drives tumor progression^41^.

### RGD molecules in the substrate increase the rate of MCA growth

**Figure 2:**
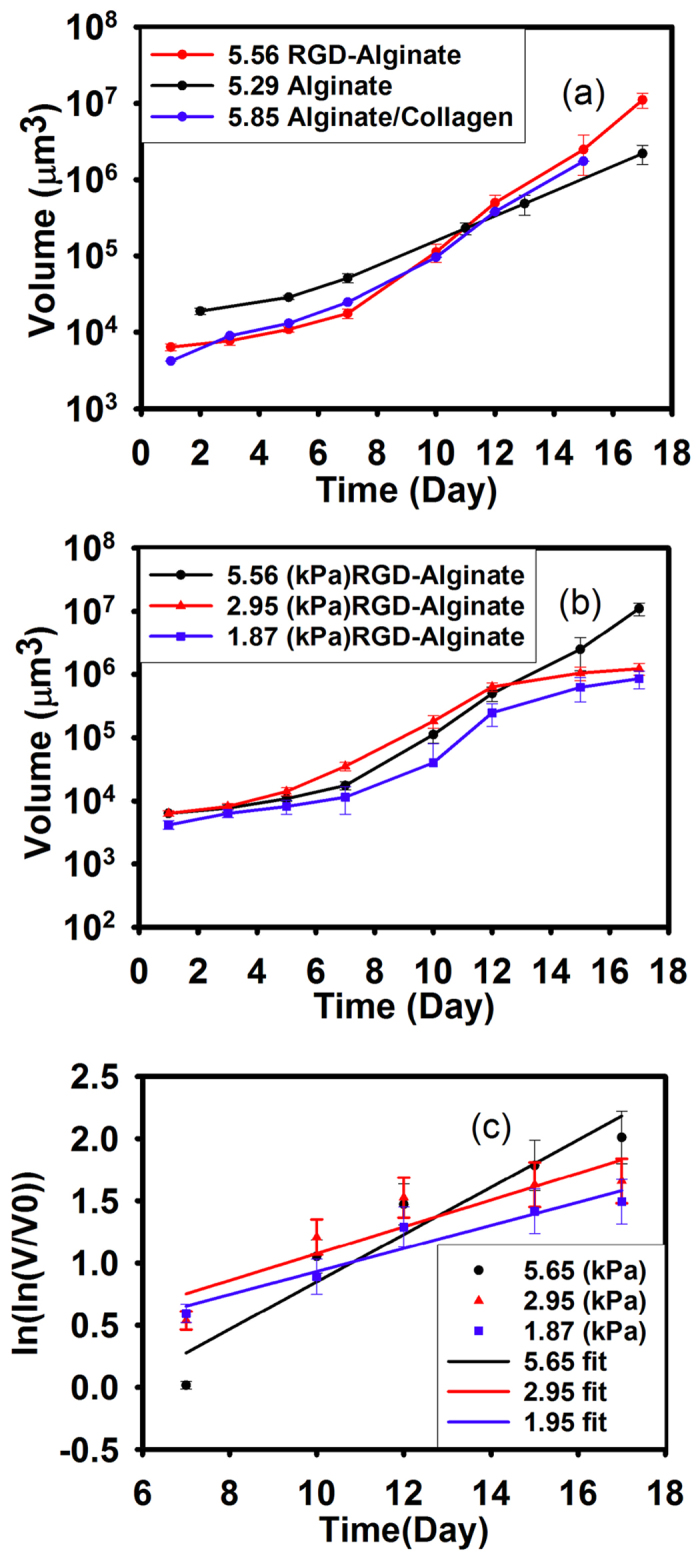
The presence of RGD leads to growth at a similar rate as collagen, which are both twice as fast as pure alginate. (**a**) Growth of M6 cells with RGD-alginate, collagen/alginate composit, and pure alginate. (**b**) Growth of M6 cells within the 5.29, 2.85, and1.85 kPa RGD-alginate gel. Mean is shown from four separate experiments, with 40 to 100 MCAs at each time point for each culture condition. Error bars are standard error of the mean. For statistical analysis we did t-test or Mann-Whitney test depending on the data distribution for each time point and P-values are between 0.3–0.6 which shows there is not a statistically significant difference in the results. (**c**) The slope of the single linear fit of the Gompertz equation can be interpreted as MCA growth rate.

To examine the roles of mechanics and biochemistry in model MCA growth, we introduce biochemical signaling and adhesive properties to the matrix by adding collagen to the alginate at a concentration described in Table 1 to obtain a matrix with the stiffness of approximately 5.55–5.85 kPa. We introduce adhesion alternatively by including a small amount of alginate conjugated with an RGD-containing peptide^36^ to obtain a final concentration of 100 fmol peptide per 1 ml of the gel. We encapsulate the transfected M6 and monitored the cell growth for 15 days. Comparing the results from these samples with the cell growth in the pure alginate of the same stiffness (Fig. 2a) shows that the trends of MCA growth are similar for all the chemical environments, but that the final average sizes of the MCAs are different for different chemical environments. The final MCA size in the presence of peptides is larger than for those without peptide, however, the increase in the average size can be seen in the collagen sample earlier than the pure alginate as well as for the RGD samples. The average MCA size in the presence of RGD approximately doubles from day 10 to 12 (from 50 ± 17 to 95 ± 25 μm) while MCAs in pure alginate appear to approximately double in size between day 13 to 17 (from 64 ± 30 to 149 ± 44 μm), suggesting that the presence of RGD may fasten MCA growth.

To clarify the relative impact of microenvironment mechanics with chemical cues in MCA growth, we fabricated gels with the same RGD concentration but with different alginate concentrations, and measured the encapsulated M6 cells’ growth for 17 days.

The trends of MCA growth in alginate with or without RGD are similar, however, the rapid growth phase occurs earlier in the presence of RGD within the substrate, as shown in Fig. 2b. Without RGD, there appears to be a lag of approximately 3 days before cell growth within the MCA rapidly increases, whereas with RGD, this growth occurs nearly immediately. The growth trends in the presence of RGD are consistent with our measurements in the pure alginate scaffold, with MCAs again growing larger within the stiffer substrate. Moreover, the growth rates determined from Gompertz’s law are faster in the presence of RGD. Values of parameter α which calculated by fitting the data with a single linear fit (r^2^ = 0.92–0.95), are 0.0927, 0.123, 0.1903 for 1.87, 2.95, and 5.56 kPa gels, respectively. Since the overall growth profile is very similar with or without RGD, this suggests that the mechanical stiffness of the environment may set the growth of MCAs, while RGD appears to expedite the growth phase.

### Substrate mechanics modulate both the shape of growing MCAs and the cell nuclei

As the MCAs grow, they compress their surrounding microenvironment, but these forces also compress the MCAs, and the individual cells and their organelles as well. In earlier studies it has been shown that MCAs tend to grow in the regions of lower stress, and thus the shape of the MCAs depends on the local solid stress field^37^,^42^. Cheng *et al*., showed that the local stress field is higher at the minor axis of the oblate MCAs from the displacement of the imbedded microbeads in the gel.

**Figure 3:**
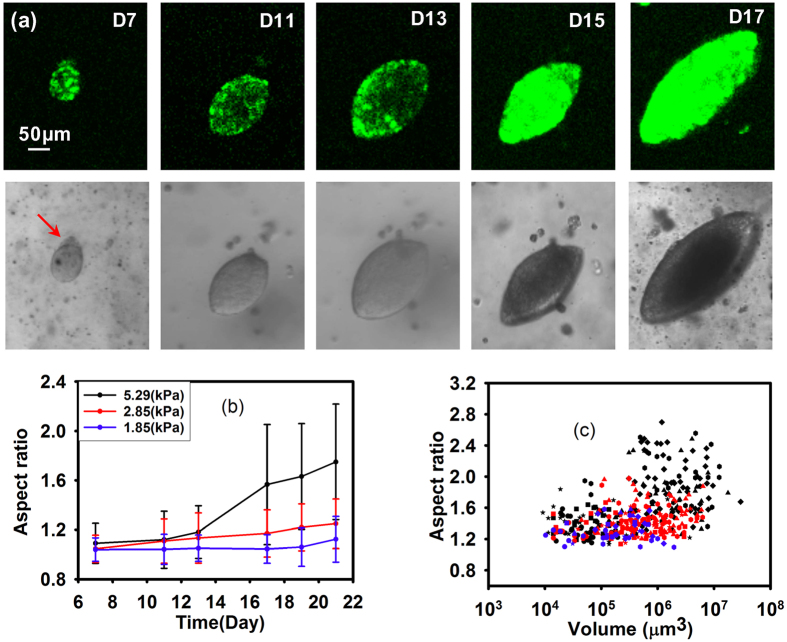
MCAs transition from spherical to elliptical shapes over time. (**a**) Fluorescence and phase contrast images from day 7–day 17. The red arrows show the beginning of the transition. (**b**) The Average aspect ratio of the MCAs at different time point in the gels of different stiffnesses over time shows that more of the MCAs in the stiffest gel (E = 5.29 kPa) are becoming elliptical. Mean is shown from four separate experiments, with 40 to 100 MCAs at each time point for each culture condition. Error bars are standard error of the mean. For statistical analysis we did t-test or Mann-Whitney test depending on the data distribution for each time point and P-values are between 0.3–0.6 which shows there is not a statistically significant difference in the results. (**c**) The elliptical shape transition in the MCA is more related to stiffness of the substrate than the volume increase of the MCAs.

These higher stresses might shift MCA growth from spherical to oblate spheroid “pancake” shapes, as shown in Fig. 3a, which has been observed previously^42^,^43^. The red arrows in Fig. 3a highlights that the proliferation and expansion defines the major axis, where the MCA stress is minimal^43^.This transition occurs in all gel stiffnesses, yet we observe that this transition to elliptical growth occurs at an earlier time point, and much more completely in the stiffest gel (E = 5.29 kPa) as compared to the softer gels. The shape factor distribution of the MCAs can be seen in Supplementary Fig. S2.

As the MCAs grow, the pressure in the stiffer gels for a given volume change is higher and influences the shape of the MCAs more.In all matrix stiffnesses the aspect ratio of the elliptical MCAs increases as the MCAs grow, however, the aspect ratio is more correlated to the stiffness of the gel than the volume of the MCAs (see Fig. 3c). Therefore, this aspect ratio transition occurs faster and to a larger extent in stiffer gels Fig. 3b.

**Figure 4:**
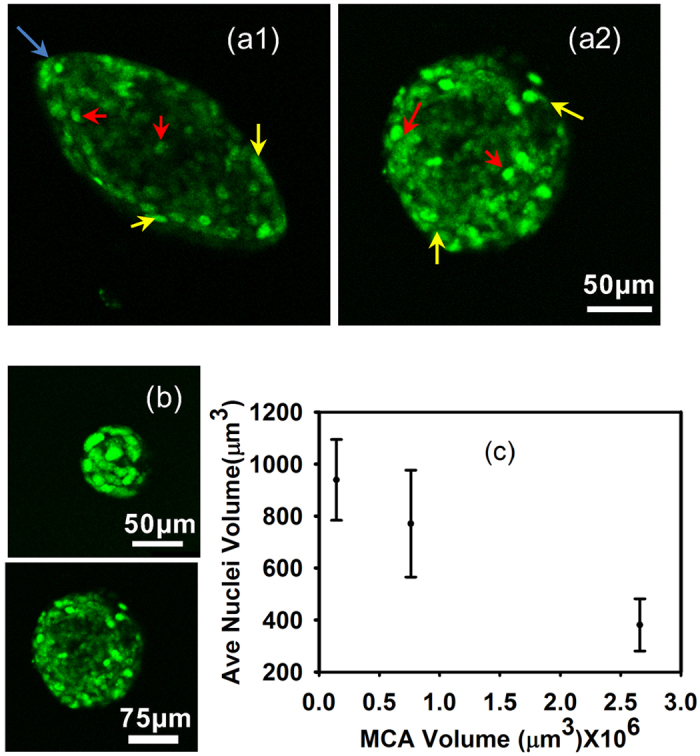
Nuclei deformation at different position of MCAs. Red and yellow arrows indicate representative nuclei at the sides, center, and the curvature of the MCAs with different sizes and shapes. Both MCA morphologies (spherical and elliptical) shown here have similar volume. (**a1**) in the elliptical MCAs, cell nuclei on the minor axis, are more elongated and they are more spherical towards the center. Blue arrows indicate the region of highest curvature. (**a2**) in the spherical MCAs there is little difference between the size of the nuclei throughout the MCA regardless of the position. (**b**) Spherical MCAs with different volume shows different nuclear size. (**c**) Nuclear volume as a function MCA volume in spherical MCAs. Means are from 10 to 15 Nuclei in each MCA; Error bars are standard error of the mean. These measurements were done for 2 to 4 MCAs with the same volume and the statistical analysis test shows there is no statistically significant difference between the inputs (P-value = 0.5–0.6).

The effects of environmental stiffness do not only make the entire MCA more elliptical, but they may also asymmetrically compress individual cells and their nuclei. By imaging the nuclei of the cells within the MCAs with confocal microscopy, we observe that nuclei in the oblate MCA differ in size and shape as a function of the position in the MCA; nuclei at the minor axis sides of the elliptical MCAs (Fig. 4a1, yellow arrows, where the curvature is lowest) are more elongated, mirroring the overall MCA shape, while those closer to the center are more spherical, as shown in Fig. 4a1. The approximate nuclear major axis diameters range from 11–14 μm and the average volume for the nuclei at the minor axis sides of oblate MCAs are around 150 μm^3^ while the average volume for the nuclei at tips (Fig. 4a1, blue arrows, in regions of highest curvature of the MCA) is approximately 321 μm^3^. However, these variations in nuclei volumes are not apparent in the spherical MCAs; nuclei diameters range from 8–9.5 μm, with corresponding volumes of 381 μm^3^, and these nuclei with different size are randomly distributed in the MCA structure (Fig. 4a2, yellow and red arrows). In both elliptical and spherical MCAs, for the bigger MCA, the nuclei volume is smaller, as shown in Fig. 4b for spherical MCAs. Moreover, the average nuclei size as a function of volume in the same MCAs is plotted in Fig. 4c, illustrating that nuclei appear more compressed in regions of higher cell density. Mechanical compression may explain the observed volume and morphological changes of the cells; the same force range which causes spherical MCAs to become more elliptical, might also compress the cells and their nuclei. Nevertheless, we cannot exclude the possibility of an unknown mechanism causing the nuclei morphology to appear compressed.

## Discussion and Conclusion

Using a highly mechanically and biochemically tunable model 3D system, we have shown that MCA properties are strongly determined by the local mechanical environment. Local mechanics regulate growth rate, cell and MCA shape, the ability of the MCA to spread, and through nuclear compression may directly affect gene expression. The presence of RGD or collagen provides a binding site for cell adhesion, and also promotes faster cell growth, and does produce larger MCAs.

Our findings are consistent with several reports finding that the matrix stiffening promotes tumor growth^3^. Conversely, other studies employing agarose gels found that stiffer 3D environments reduce cell growth^37^; however, they used stiffnesses ranging from E = 216 Pa to E = 367 Pa, much less than the stiffness of breast tissue found both in normal and metastatic environments, potentially placing it outside a directly comparable range; moreover, previous studies have reported that agarose is not a suitable material for studying mechanical substrate interactions due to several drawbacks^21^,^22^. The main differences between these diverse studies lie in the mechanical properties of the substrate and the cell, failure mechanism of the matrix and also the structure of the microenvironment^44^. Moreover, cancer cell proliferation is known to be very cell-type dependent^45^ which may also lead to differences in results.

Our study shows that the increased scaffold stiffness directs the shape of a growing MCA, consistent with previous studies^42^. We also find that the transition from spherical to elliptical occurs over time, yet that in stiffer scaffolds it is accelerated and occurs earlier. This suggests that the MCA shapes and local proliferation may be set by a given stress, possibly at which the alginate mechanically yields, which can occur in a range of scaffold stiffness, but is reached more quickly in stiffer gels. This may be due to the same strain or volume change inducing a higher stress or pressure in the stiffer gels, so that the yield stress will be reached and promote symmetry-breaking. Since alginate is ionically crosslinked, it can reform after yielding, creating a mechanically robust gel.

In spherical MCAs, cells throughout the cluster have similar volumes and shapes, as do their nuclei, and are thus likely experiencing relatively similar and isotropic stresses throughout the MCA. In contrast, we observe a variety of cell morphologies in the elliptical MCAs: nuclei of cells are substantially smaller, and are compressed along the minor axis of the cell cluster, and resemble the overall MCA shape. The elliptical structure of the cluster, the constituent cells, and their nuclei suggest that the environmental forces are strongly asymmetric. This location-specific cell and nuclear compression provides a unique force signature for different positions in the MCA, and thus may also provide an avenue for mechanotransduction feedback^46^.

Future work will examine how ubiquitous this behavior is in diverse cancers, and may contribute to novel mechanical strategies for treatment or confinement of MCAs or tumors *in vivo*.

## Additional Information

**How to cite this article**: Khavari, A. *et al*. Composite alginate gels for tunable cellular microenvironment mechanics. *Sci. Rep*. **6**, 30854; doi: 10.1038/srep30854 (2016).

## Supplementary Material

Supplementary Information

Supplementary Movie S1

Supplementary Movie S2

Supplementary Movie S3

Supplementary Movie S4

## Figures and Tables

**Table 1:** Alginate and alginate blends with collagen and RGD-alginate shear and young moduli with different concentration in cell growth medium.

Polymer (Matrix)	Alginate	Alginate	Alginate	Alginate/Collagen	Alginate/RGD-alginate	Alginate/RGD-alginate	Alginate/RGD-alginate
Concentration	2 w/v%	1 w/v%	0.5 w/v%	0.75 w/v% /0.1 mg/ml	2 w/v% /100 fmol/ml	1 w/v% /100 fmol/ml	0.5 w/v% /100 fmol/ml
Complete gelation time (hr)	4	5	6	2.5	4	4.5	5
Shear modulus (kPa)	2.15 ± 0.15	1.16. ± 0.17	0.753 ± 0.08	2.35 ± 0.15	2.26 ± 0.2	1.2 ± 0.1	0.76 ± 0.089
Young modulus (kPa)	5.29 ± 0.25	2.85 ± 0.25	1.85 ± 0.08	5.8 ± 0.35	5.56 ± 0.25	2.95 ± 0.25	1.87 ± 0.08
