# Corrigendum: Composite alginate gels for tunable cellular microenvironment mechanics

**DOI:** 10.1038/srep32905

**Published:** 2016-09-08

**Authors:** Adele Khavari, Magnus Nydén, David A. Weitz, Allen J. Ehrlicher

Scientific Reports 6: Article number: 3085410.1038/srep30854; published online: 08 03 2016; updated: 09 08 2016

In this Article, David A. Weitz is incorrectly affiliated with ‘Department of Bioengineering, McGill University, Montreal Canada H3A 0C3.’ The correct affiliation is listed below:

School of Engineering and Applied Sciences, Department of Physics, Harvard University, Cambridge, Massachusetts 02138, USA.

