# Supplementary material for: Composite alginate gels for tunable cellular microenvironment mechanics
Source: Sci Rep. 2016 Aug 3;6:30854. doi: 10.1038/srep30854 (PMC4971458; doi:10.1038/srep30854)
Supplement: Supplementary Information [file srep30854-s1.pdf]

Composite alginate gels for tunable cellular microenvironment mechanics

Adele Khavari[1,2*], Magnus Nydén [1,3], David A. Weitz[4], Allen J. Ehrlicher[4,5,6,7*]

[1] Applied Chemistry, Chemical and Biological Engineering, Chalmers University of Technology, SE-412 96 Göteborg, Sweden.
[2] SUMO Biomaterials VINN Excellence Center, Chalmers University of Technology,
SE-412 96 Göteborg, Sweden.
[3] UCL Australia , 220 Victoria Square, Adelaide, SA 5000Australia
[4] Department of Bioengineering, McGill University, Montreal Canada H3A 0C3.
[5] School of Engineering and Applied Sciences, Department of Physics, Harvard University, Cambridge, Massachusetts 02138, USA.
[6]Beth Israel Deaconess Medical Center, Boston, Massachusetts 02115, United States
[7]Harvard Medical School, Boston, Massachusetts 02115, United States
*Authors to whom any correspondence should be addressed.
E-mail:allen.ehrlicher@mcgill.ca; adele@chalmers.se

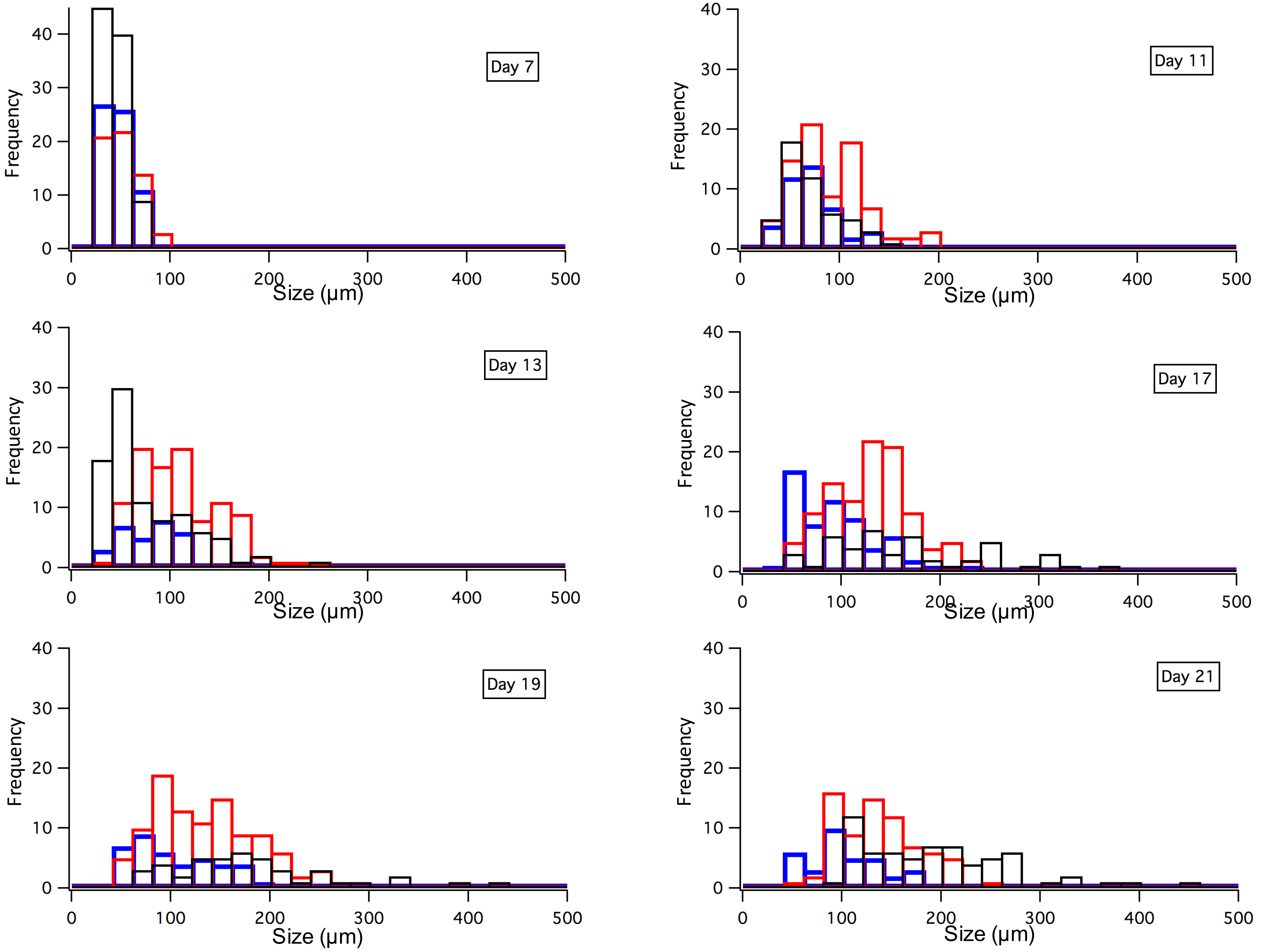

Fig S1. Size distribution of the MCAs at different days. Black, red, and blue are different elastic moduli, 5.29, 2.85, and 1.85 kPa.

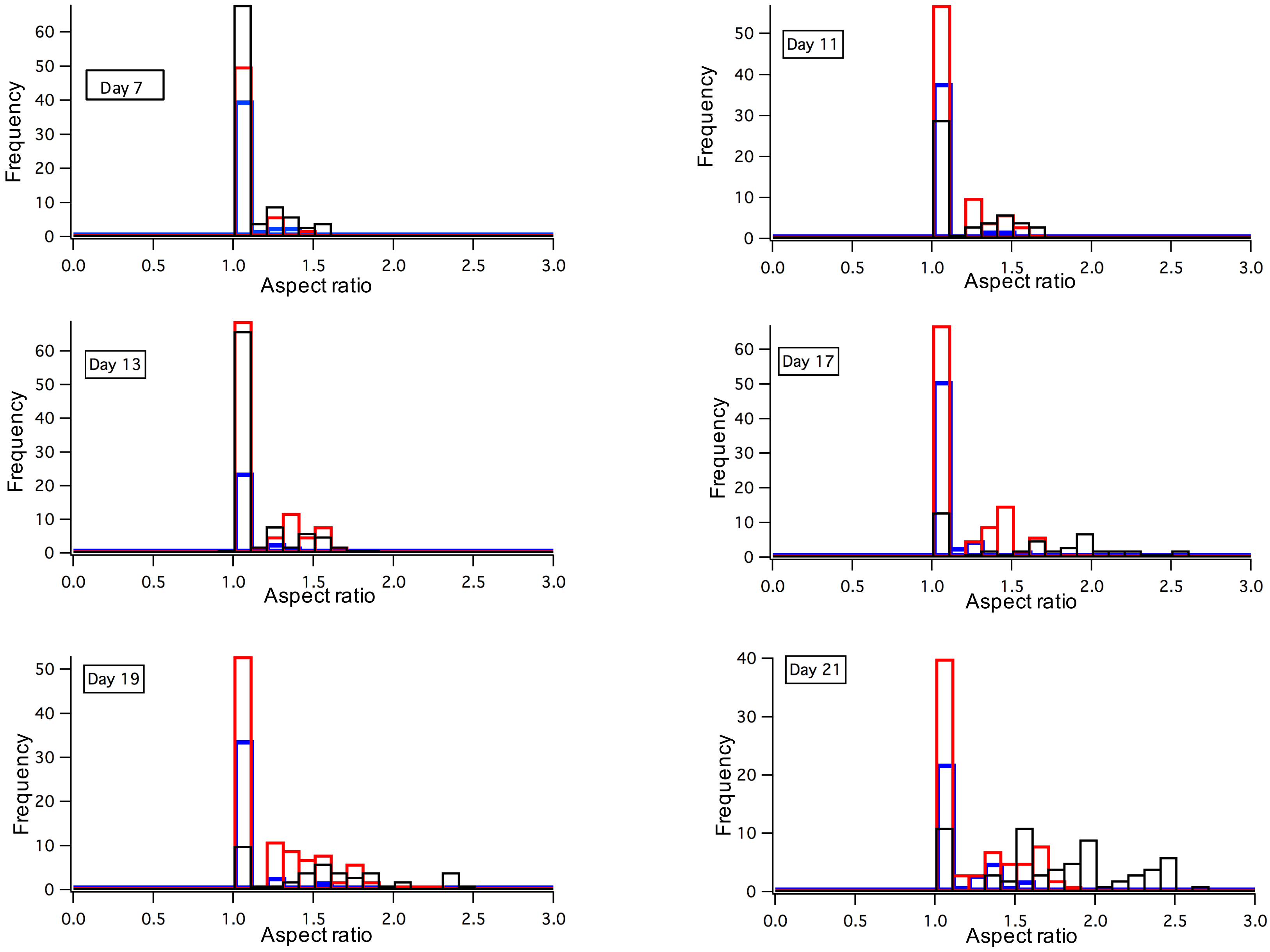

Fig S2. Shape factor (aspect ratio) distribution of the MCAs at different days. When the difference between major and minor axis is less 5%, the MCAs with an aspect ratio of one are considered to be spherical. Black, red, and blue are different elastic moduli, 5.29, 2.85, and 1.85 kPa

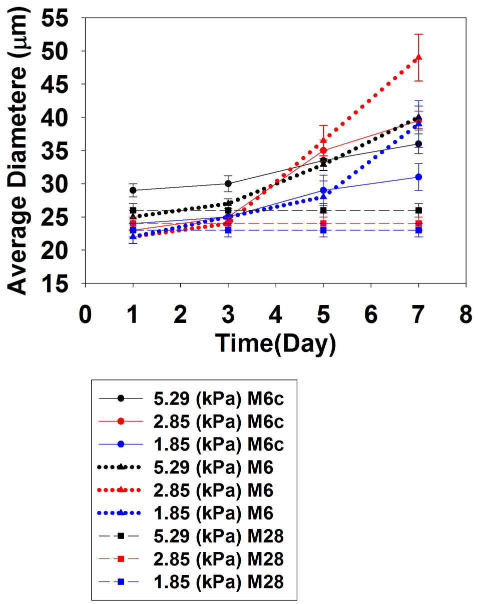

Fig S3. Average diameter of MCA’S of different cell lines in different elastic moduli. Black, red, and blue are different elastic moduli 5.29, 2.85, and 1.85 kPa.

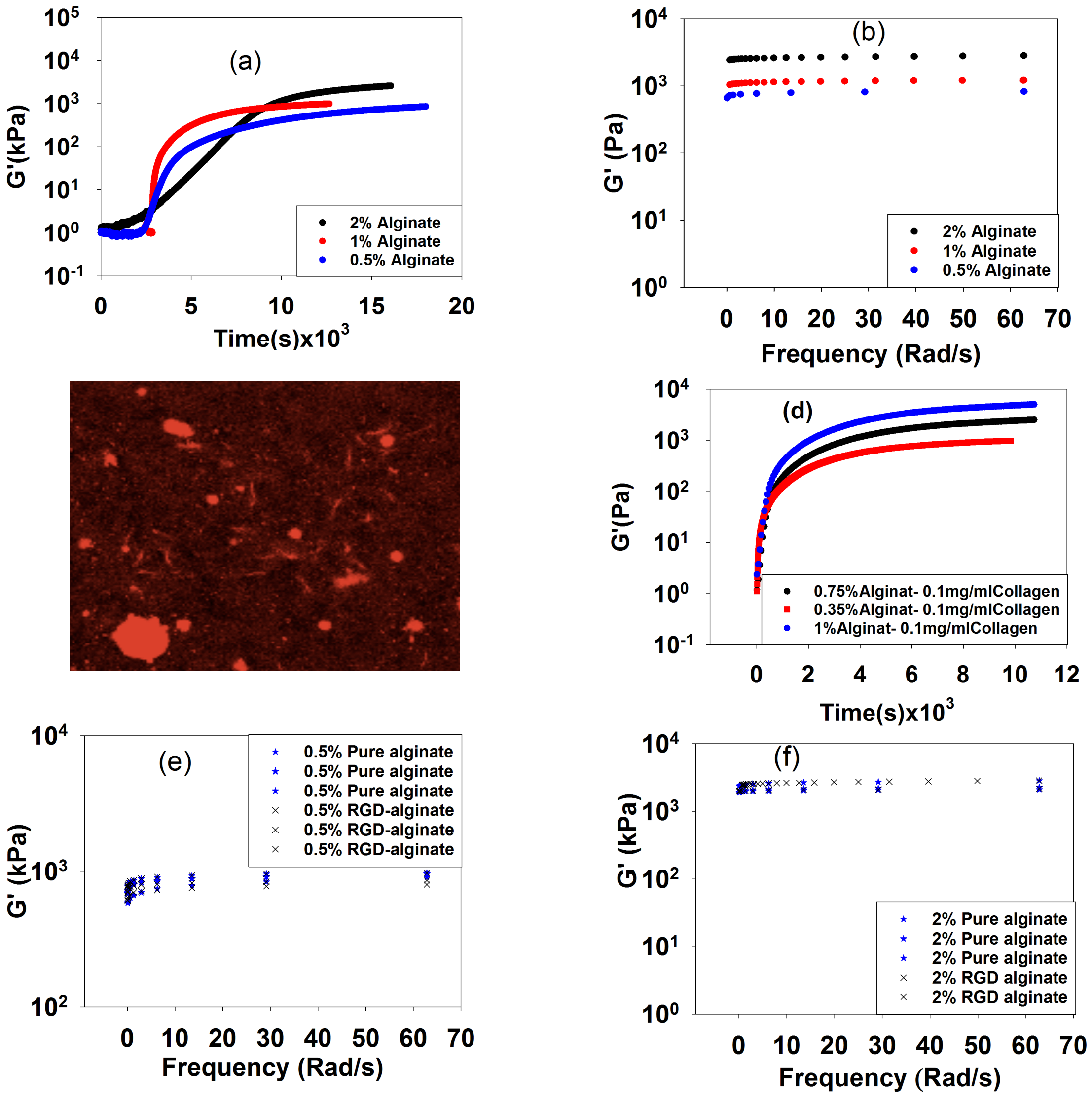

Fig S4. (a) Time sweep measurements of alginate, (b) Frequency sweep measurements of three different concentration of alginate. (c) Fluorescence image of collagen fiber and cells after 15 days of culture. (d) Time sweep measurements for different composition of alginate and collagen. (e), (f) frequency sweep measurements for 0.5%, and 2% alginate.

Supplementary movies

- S5. Z-stack images of MCA’s in the gel with elastic modulus of 2.85 kPa
- S6. Z-stack images of MCA’s in the gel with elastic modulus of 5.29 kPa
- S7. Z-stack images of a single MCA in the gel with elastic modulus of 2.85 kPa
- S8. Z-stack images of a single MCA in the gel with elastic modulus of 5.29 kPa
